# All Tapped Out: Touchscreen Interactivity and Young Children’s Word Learning

**DOI:** 10.3389/fpsyg.2017.00578

**Published:** 2017-04-12

**Authors:** Colleen Russo-Johnson, Georgene Troseth, Charlotte Duncan, Almaz Mesghina

**Affiliations:** Psychology and Human Development, Vanderbilt University, NashvilleTN, USA

**Keywords:** educational technology, touchscreen, app, interactive, tap, drag, haptic exploration, executive function

## Abstract

Touchscreen devices differ from passive screen media in promoting physical interaction with events on the screen. Two studies examined how young children’s screen-directed actions related to self-regulation (Study 1) and word learning (Study 2). In Study 1, 30 2-year-old children’s tapping behaviors during game play were related to their self-regulation, measured using Carlson’s snack task: girls and children with high self-regulation tapped significantly less during instruction portions of an app (including object labeling events) than did boys and children with low self-regulation. Older preschoolers (*N* = 47, aged 4–6 years) tapped significantly less during instruction than 2-year-olds did. Study 2 explored whether the particular way in which 170 children (2–4 years of age) interacted with a touchscreen app affected their learning of novel object labels. Conditions in which children *tapped* or *dragged* a named object to move it across the screen required different amounts of effort and focus, compared to a non-interactive (*watching*) condition. Age by sex interactions revealed a particular benefit of dragging (a motorically challenging behavior) for preschool girls’ learning compared to that of boys, especially for girls older than age 2. Boys benefited more from watching than dragging. Children from low socioeconomic status families learned more object names when dragging objects versus tapping them, possibly because tapping is a prepotent response that does not require thoughtful attention. Parents and industry experts should consider age, sex, self-regulation, and the physical requirements of children’s engagement with touchscreens when designing and using educational content.

## Introduction

“It’s already a revolution, and it’s only just begun.” With these words, the Apple iPad was launched on the world in 2010, a few years after families first fell in love with miniature touchscreens on their phones. Today, mainstream adoption of smartphones has permeated the socioeconomic divide in the U.S. (Smith, 2013), with most families of all income levels now having a touchscreen device. According to a 2015 study, 90% of toddlers in a low-income, traditionally underrepresented population in the U.S. had used a touchscreen by age 2, and 83% of children under 5 had a tablet computer in their home (Kabali et al., 2015). As touchscreen devices and apps (applications) quickly became a part of daily life for youth and adults, the developmental and educational effects of interactive media emerged as an important, highly debated topic. Yet research into the effectiveness of touchscreens for children’s learning and their impact on family life has lagged behind (Troseth et al., 2016).

Despite the lack of research, parents and teachers avidly buy touchscreen apps. Approximately, 80% of the over 170,000 educational apps in the iTunes store (Apple, 2017) are designed for children, with toddlers and preschoolers representing the most targeted age group (Shuler, 2012). Because children are given access to touchscreens at a very young age, it is imperative to study the effects of interactive technology on children’s learning and development.

Scientific investigation of screen media for children is not new. Decades of research document both positive and negative effects of exposure to children’s television (see Anderson and Kirkorian, 2015, for a review). Such effects are highly dependent on the content of the media and the viewing context (i.e., viewing with or without an adult co-viewer), as well as the age of the child. Research has consistently demonstrated that young children, particularly those under 24 months of age, learn better from an individual who is with them in person rather than on a screen (Barr, 2010; Troseth, 2010). For instance, in one study, 15- to 24-month-old toddlers learned significantly fewer words from a children’s television program compared to learning directly from an adult; very few children under 22 months learned any words from the TV program (Krcmar et al., 2007). The same pattern of results has been found in infants’ and toddlers’ problem solving and imitation of behaviors modeled on video or observed “face to face” (Troseth and DeLoache, 1998; Barr and Hayne, 1999; Schmitt and Anderson, 2002).

However, this learning difference might have less to do with the video medium than with the relatively passive nature of watching events on a TV screen (Christakis, 2014). Recent advances in technology allow viewers to actively engage with people on a screen via video chat (e.g., Skype and FaceTime). Research indicates that children as young as 24 months successfully use video as a source of information when an adult on screen interacts with them in socially contingent ways (Troseth et al., 2006; Roseberry et al., 2014; Myers et al., 2016). Thus, active engagement with a responsive on-screen partner seems effective in helping young children to learn from video.

Touchscreen devices afford a different, non-social kind of contingent interaction: physical touch leading to an on-screen response. Individual differences in motor skills, dexterity, and decision making now play a role in how a medium is experienced. Physical interactivity with the screen gives the user agency as he or she chooses and directs what happens. For example, one child may tap an object on the screen that then displays an animation; a child who does not tap the object will have a different experience. These contingent interactions can create an adaptive, scaffolded experience for the user, which is a powerful aid for learning when properly designed (Hirsh-Pasek et al., 2015). In essence, the individual child’s characteristics, preferences, and actions mold the medium, within the bounds of the interactive design. Based on the idea that physical, like social, contingency will make screen media better for learning, developmental researchers are now exploring this new technology (e.g., Choi and Kirkorian, 2016).

In prior studies that are relevant to the subject of learning via touchscreen interactivity, adults and children acted on objects, or actively engaged with material on a computer, compared to merely viewing the same objects or events. Important differences in these situations include additional information available from touch compared to watching (Smith and Olkun, 2005; Bara et al., 2007; Kalenine et al., 2011; Möhring and Frick, 2013), and the fact that active engagement in an experience changes how the event is processed and remembered (James and Swain, 2011; Kaplan et al., 2012; Kersey and James, 2013). The type of interactive behavior and its temporal and spatial correspondence to on-screen events also may affect memory and learning (Sapkota et al., 2013; Schwartz and Plass, 2014; Choi and Kirkorian, 2016; Kirkorian et al., 2016).

Much early learning happens through multisensory exploration, which becomes more sophisticated and efficient across the preschool period (Thelen, 2000; Scofield et al., 2009). The combination of *visual information* and *touch* (visuo-haptic interaction) promotes young children’s learning over simply watching. For example, preschoolers offered 3D shapes were more likely to manually explore the shapes and recognize them later than were children given 2D paper cutouts of the shapes (Kalenine et al., 2011). Similarly, low-SES kindergarteners who explored target letters both visually and haptically had significantly improved reading skills compared to those who only explored the letters visually (Bara et al., 2007). The same advantage of visuo-haptic exploration was found for babies: 6-month-olds *mentally* rotated objects with greater success if they first *manually* explored the objects by hand, compared to those who only observed the objects rotating (Möhring and Frick, 2013).

Carrying out actions on objects changes brain responses when perceiving the stimuli later. After physically engaging in actions with objects (versus observing someone else do the actions), children between the ages of 5 and 7 demonstrated greater neural activity in motor and sensorimotor brain areas while perceiving the stimuli (*seeing* the objects or actions, or *hearing* verbs describing the actions – James and Swain, 2011; Kersey and James, 2013).

Even indirect physical interactions with on-screen objects using a computer mouse or stylus benefit learning. In one example, 9-year-olds and college students explored a series of 2D shapes on a computer screen by either manually dragging each object with a mouse to rotate it (interactive condition) or observing the object rotate automatically. During a later test, participants in the interactive condition mentally rotated significantly more objects (Smith and Olkun, 2005). In another study, adults performed better in immediate and 3-week-delayed recall and recognition tests after they dragged target objects with a computer mouse, compared to clicking on objects that then moved automatically (Schwartz and Plass, 2014). Dragging a virtual object (e.g., an eraser, a paintbrush) in this study was an “iconic” movement related to the meaning of the to-be-learned phrase and the depicted context (e.g., *erase the blackboard*; *paint the fence*). Enacting a meaningful action promoted better memory than did clicking to produce a very similar object movement. In general, active manipulation can make an experience with digital technology “minds-on” for both adults and children, increasing their cognitive engagement and supporting learning (Hirsh-Pasek et al., 2015).

The spatial correspondence of a user’s physical interaction with an on-screen object or event can also promote learning, but results with young children are not straightforward. Adults’ short-term memory for stimuli improved when their touchscreen taps with a stylus corresponded with the location of the target (Sapkota et al., 2013), which directed participants’ attention to the to-be-remembered information. In contrast to this clear finding, emerging research with preschool-aged children reveals an intriguing paradox. Children younger than 30 months of age reliably learned new object labels when they had to tap the on-screen box where a named target object was hidden (specific contingency), but they did not learn when they tapped elsewhere on the screen (Kirkorian et al., 2016). However, toddlers over 30 months learned better when only general tapping was required or if they simply watched events unfold; for them, the requirement to tap in the relevant spot actually detracted from learning. Choi and Kirkorian (2016) found the same results with a different learning task, except the children who were helped by specific contingency were 6 months younger than the comparable group in the previous study. Therefore, the particular kind of contingent screen interaction that aids learning may differ depending on the task and the age of the learner.

The fact that interaction can direct children’s attention could be a powerful tool for learning, but it could also create distraction, depending on the design of a touchscreen activity and individual differences in children. Interactive elements such as hotspots and games appear in the majority of electronic books (e-books) that young children “read” on touchscreens, but such features often are unrelated to the story (Guernsey et al., 2012). In a recent meta-analysis, *multimedia* features (such as animations and sound effects) that directed attention to the e-book story enhanced learning, but interactive games, pop-ups, and hotspots (whether story-relevant or -irrelevant) detracted from young children’s learning, especially for children from disadvantaged backgrounds (Takacs et al., 2015). Similarly, manipulative elements in print books (pull tabs, flaps, textures) have been shown to impede word learning in 20- to 36-month-old children (Chiong et al., 2010).

Two important results emerged in a recent study using a simple e-book in which a narrator labeled pictured objects (Strouse and Ganea, 2016). First, toddlers (19–23 months) learned a word when they had to tap on the object to go to the next page, but did not learn if the story progressed automatically; thus, simple, on-task interaction promoted learning. Second, children failed to learn when their touch produced a rewarding, child-friendly (but irrelevant) sound effect and animation before the page turned. Thus, even simple off-topic interactivity appeared to interfere with learning.

Why is screen interactivity such a double-edged sword for very young children? Numerous research studies indicate that young children have immature executive functions, such as the ability to focus attention and control their impulses (Carlson, 2005; Garon et al., 2008; Richter and Courage, 2017). For instance, 2- and 3-year-old children’s ability to push one of two buttons to complete a spatial matching task related to measures of their inhibitory control (assessed with Kochanska’s *snack delay* task), although few young 2-year-olds had sufficient inhibitory ability to complete either task (Gerardi-Caulton, 2000). In another study (Diamond and Taylor, 1996), children had to inhibit a response tendency to match an adult’s behavior: if a researcher tapped a peg on the table once, the child was to tap the peg twice (and vice versa). Three-year-olds did not even pass a pre-test to show understanding of the rules; of children 3.5 years and above, more girls than boys passed the pretest. In the actual test, children started out complying, but younger children could not sustain their attention over time (becoming both faster in responding and less accurate). Although 3-year-olds in another study were to be rewarded for pointing to a box they knew was empty (rather than a box they knew contained candy), they could not inhibit pointing to the baited box (Russell et al., 1991).

According to longitudinal research, impulse control (complying with an adult’s rule) is especially challenging for toddlers (particularly for boys), with inhibitory ability developing across the preschool years (Kochanska et al., 1996). Therefore, when using an e-book or touchscreen app, the need to disengage from an interactive element and re-focus attention on the story or educational content might challenge young children’s limited ability to regulate their attention and actions (Fisch, 2000; Mayer and Moreno, 2003; Sweller, 2005; Bus et al., 2015). Research on children’s developing inhibitory control suggests that interactivity must be used strategically, especially with very young children, to promote learning rather than distraction.

For our app, we chose word learning as an age-appropriate task that would require children’s attention. Increasing children’s vocabulary is a focus of numerous commercially available apps, and research indicates that preschoolers can “fast map” the association between an object displayed on a screen and its label. For instance, 2-year-olds in one study saw the image of a known object (e.g., a ball) and a novel object on side-by-side computer screens, and a voiceover ambiguously asked them to point to the “glark” (Spiegel and Halberda, 2011). Only by process of elimination (following the “mutual exclusivity” principle) could they figure out the word’s referent. After six trials on which they were asked to figure out and point to different named novel objects, they saw all six novel objects simultaneously and were able to pick out whichever one the researchers named. In another study, 2- and 3-year-olds saw an image of an unknown target object in the middle of computer screen while a narrator offered a novel label several times. Then the target disappeared and four images (the target and three unfamiliar foils) appeared together on the screen (Scofield et al., 2007). Children selected the named object at rates above chance, and above the rate at which they selected a non-labeled target (used as a familiarity control). In numerous studies using the preferential looking paradigm, preschoolers have also shown that they can learn the association between an object on a screen labeled in a voiceover and its name (Golinkoff et al., 1987; Schafer and Plunkett, 1998; Werker et al., 1998). Identifying a named object on the screen is the kind of response that would be considered “word learning” in terms of an app, although language researchers distinguish between evidence for initial learning of a word-object mapping and long-term retention of word meaning (Werker et al., 1998; Horst and Samuelson, 2008; Axelsson and Horst, 2013; Bion et al., 2013).

If interactivity is successful and children do learn from a touchscreen, an equally important issue is whether they can apply, or *transfer*, what they learn to the world outside the screen (Barr, 2010, 2013). In an early study using a touchscreen, 15-month-old toddlers failed to transfer a behavior they reliably learned on the screen (pushing a virtual button on a firetruck to elicit a siren sound) to a real toy, or from the toy to the screen (Zack et al., 2009). Older children have been more successful at transfer; for instance, in a study with 27- to 35-month-old preschoolers, researchers pointed out the similarities between the same scene on a touchscreen and on a felt board (a bear and four distinctive 2D hiding places). Then children either were told to touch the bear on the touchscreen so that it would hide, or to watch the bear hide automatically. When children were asked to find the bear on the felt board (that is, to transfer information from the virtual scene), they were more successful in the interactive than the watching condition (Choi et al., 2016). In another study, 30- and 36-month-olds saw videos on a computer screen of puppets hiding in another room, and then were asked to go to the room and find the puppets. Those who pressed a button on the computer to play each “hiding” video more often found the puppet, compared to children who watched the hiding on a non-interactive video, again showing the value of simple, on-task contingency (Lauricella et al., 2010). Furthermore, after 4- to 6-year-old children rearranged a set of virtual rings on pegs on a touchscreen to solve a Tower of Hanoi strategy problem, they solved an analogous 3D problem with real pegs and rings, demonstrating real-world transfer (Huber et al., 2015).

In the research reported here, we examined how children of preschool age physically interact with touchscreen media, and how different types of contingent screen interactions impact children’s learning of novel object labels. In Study 1, we designed an app that purposely included “unsupportive, incidental, inconsiderate hotspots” (Zucker et al., 2009) in a tap-the-butterfly filler task that was irrelevant to the main word-learning task. In this preliminary research, we observed how girls and boys physically engaged with the app, focusing on children’s tendency to tap on the screen and their ability to inhibit tapping to listen to the narration. Because controlling attention and behavior is especially challenging early in development (Kochanska et al., 1996; Garon et al., 2008), we included Carlson’s (2005) snack delay task, an age-appropriate toddler measure of self-regulation, when testing our youngest age group (2-year-olds). We also recruited older preschoolers to use the app, and compared their tapping and word learning to that of the 2-year-olds. In Study 2, we incorporated “supportive, considerate” (Zucker et al., 2009) interactivity – designed to support learning – into a new app and purposely excluded from the design any “inconsiderate” hotspots. We asked children to actively engage with (virtual) novel objects on the screen in a way that might direct attention to them during a naming task. We looked at the connection between different levels of interaction (i.e., *dragging*, *touching*, or *watching* an object move on screen), children’s learning of the virtual object’s name, and their transfer of the name to the real, depicted 3D object. The results of the two studies provide initial information about how the affordances and design of touchscreen apps may interact with child characteristics to promote or hinder learning.

## Study 1

### Method

#### Participants

Seventy-seven typically developing, monolingual English-speaking children from a southeastern city in the U.S. participated in this preliminary study using a lab-made touchscreen app. Thirty 2-year-old children (17 males) ranged in age from 23.3 to 35.9 months (*M* = 29.4 months; *SD* = 3.5 months). Additionally, 47 older children (22 males) who came to the lab for other studies played the app (ages 46.1–72.6 months), including a group of 4-year-olds (*N* = 22; *M* = 53.9, *SD* = 3.3), and a group of 5-year-olds (*N* = 25; *M* = 66.6, *SD* = 3.8). Participants were recruited through state birth records and their parents were contacted by telephone. The majority were Caucasian and from middle-class homes. The studies reported here were approved by the university’s institutional review board, and written parental informed consent/verbal child assent was obtained.

#### Materials

We created a touchscreen word learning app using a customizable flashcard app program and displayed it on an 9.5 inch × 7.3 inch (24.1 cm × 18.5 cm) iPad tablet screen. Following the convention in many word learning studies, we included four novel object-label learning trials, as children of this age can learn up to four words per day (Axelsson and Horst, 2013).

**FIGURE 1:**
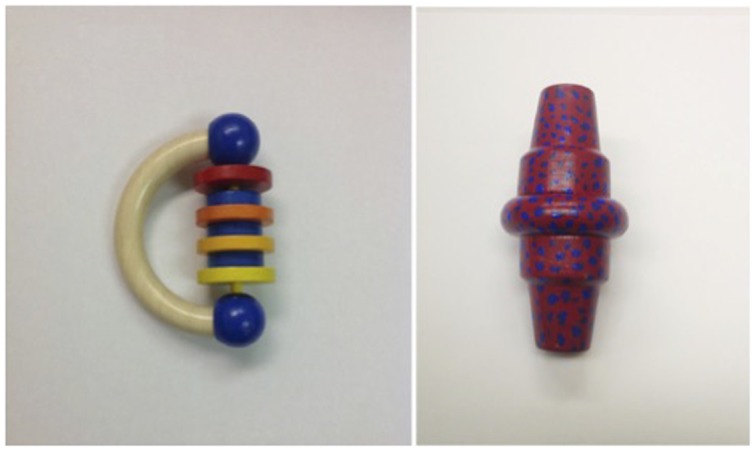
**Examples of novel objects for Study 1**.

The objects appearing in the app were painted wooden toys for which children in previous research did not have names, and similar lab-crafted objects (see **Figure 1** for one object pair). Familiar objects for practice trials on the app were small plastic toy animals. A clear plastic cup, a circular black placemat, and goldfish crackers were used for the self-regulation snack task completed by the youngest age group.

#### App Design

The app began with brief narration instructing children not to tap the screen until the voice stopped. The app was designed so it would not advance until labeling finished, no matter how many times children tapped. We made this design choice for two reasons: it ensured that all children heard the objects labeled the same number of times, and allowed us to assess how many times children tapped on the screen during object labeling, despite instructions to the contrary. After word learning trials, to maintain engagement, a screen appeared on which children could tap to make butterflies fly off, followed by a rewarding chime sound.

#### Procedure

Data were collected in a lab room furnished with a couch, chair, and low table. An experimenter interacted with children, and an assistant recorded the data during the session. Sessions also were videotaped for later coding.

The 2-year-old children first completed Carlson’s (2005) toddler snack self-regulation task (based on a measure developed by Kochanska et al., 2000). The task consists of five trials, each escalating in length, wherein the child was presented with a goldfish cracker under a clear plastic cup and told to wait until the bell was rung to retrieve the cracker. In Carlson’s instructions, the task ends when children fail a trial by retrieving/eating the treat before the bell rings, or ringing the bell themselves. We used a criterion to create a pass/fail format: children who waited until the bell for all five trials were credited with “high” self-regulation. Those who failed one or more trials were categorized as having “low” self-regulation. We were not able to collect self-regulation data for five participants. Children in the older age groups played the word-learning app after taking part in an unrelated lab task for a separate study, and therefore did not complete a self-regulation task.

Children were introduced to the iPad tablet. The child sat on the couch next to the experimenter and the parent sat nearby in a chair. The assistant watched from behind the child’s shoulder to record the data. The tablet sat on a stand on the table in front of the child. The experimenter helped children practice how to touch the iPad screen, making sure they could tap using the pad of their finger so that the device would register the interaction. During this practice, a square on the tablet screen changed color when children successfully tapped.

Next, the experimenter told participants that they were going to play a game, and opened the lab-created word-learning app that taught four novel object labels and took approximately 8 min to complete, depending on how quickly children tapped to advance between screens. Parents were asked to stay silent to enable us to see how children would respond to the app on their own. The experimenter tapped the screen to start the app, which initiated voiceover verbal instructions by a narrator. To familiarize children with the word learning game, the first of a pair of familiar toy animals (cow and elephant) appeared in the center of the screen, one at a time, and each animal was labeled five times by the narrator. After each had been labeled, children had to tap the screen to advance the app; if they did not do so spontaneously, the experimenter encouraged them to “keep playing.” On the next screen, the two animals appeared together in different locations than on the labeling slides and the narrator asked the child to touch a specific named animal. Because familiar objects trials served to teach children how to play the game, the children’s responses were not analyzed.

After another familiarization trials with a different set of animals (horse and sheep), children advanced to the actual test trials. A target (named) toy or distractor (un-named) toy appeared in the center of the screen (the other toy appeared on the next screen) and the narrator commented on it five times in a voiceover. Narration for the target object included a novel label (dax, fep, blik, or zav) and various carrier phrases spoken in child-directed speech: “Here’s the fep! Look at the fep! See the fep? Isn’t the fep neat? This is a fep!” Narration for the distractor object included the expression “this one” instead of a label, but was otherwise identical. Then the target and distractor objects appeared together on the test screen and children were asked to “Touch the fep.” Object pairings (such as in **Figure 1**) were kept consistent, but the order in which the pairs appeared, and which item of a pair was the target, were counterbalanced across participants. Whether the target or distractor object appeared first, and the location of the target item on the test screen, were counterbalanced across trials. Together, object labeling and children’s response on the test screen (for two practice and four actual trials) lasted approximately half a minute per trial.

Between word learning trials, children were presented with a series of three cocoons on the screen. Each time the child tapped a cocoon, a butterfly would appear and fly away with the narration “1”, then “2”, then “3”, followed by a rewarding chime noise. This interactive filler task was included to help maintain engagement and make the lab-created app more similar to a commercial app. Children took approximately 1 m to complete each filler task. Halfway through the word learning trials, we gave children a short break, during which they played with toys for about a minute before completing the final two word learning app trials.

#### Scoring

Tapping during the introduction and while the novel objects were being labeled was considered “taps during instruction.” Taps on the butterflies were considered “filler taps.” Trained researchers counted all taps from video of the session, and 26 videos were double coded, with excellent inter-rater reliability (Krippendorf’s alpha 0.982, with a 95% confidence interval of 0.972 to 0.991). Children’s choice of object (either their tap on or point at an object) on the word learning trials was coded during the session by the assistant and from videotape by a second coder. The few discrepancies between coders (notation mistakes made during sessions) were settled by a third coder, resulting in 100% agreement for all participants.

### Results and Discussion

#### Two-year-old Children

Across the 30 younger children, the mean number of taps during instructions and labeling was 19.2 (*SD* = 17.0), ranging from 0 to 63 taps. According to our criterion for passing the snack delay task, half of the participants were classified as having low self-regulation. We first examined relations between children’s tapping on the tablet screen during the app and their self-regulation classification. There were no differences in tapping during the butterflies filler task based on children’s self-regulation classification. In contrast, group differences emerged in the number of taps during the “instruction” portions of the app (the initial instructions and the labeling events) when children were instructed not to tap: the 13 children classified as having low self-regulation tapped significantly more (*M* = 27.5 taps, *SD* = 20.0) than the 12 children with high self-regulation [*M* = 12.1, *SD* = 12.3; *t*(20.2) = 2.34, *p* = 0.029, *d* = 0.96] (degrees of freedom adjusted from 23 to 20.2 due to unequal group variances – Levene’s test, *F* = 5.58, *p* = 0.027). On average, children who scored lower in self-regulation tapped more than twice as often as their peers during the instructions and labeling. Importantly, these were the parts of the app when children needed to focus attention on the narrator’s words. For children with high self-regulation, being able to inhibit tapping allowed them to concentrate on the instruction. The fact that children tapped equally often during the “butterflies” filler portions regardless of self-regulation classification suggests that those with higher self-regulation were equally interested in tapping, but used inhibitory control when the narrator was providing instruction.

A similar pattern was found comparing the tapping behavior of males and females. Boys tapped significantly more during the instruction portions of the app (*M* = 26.5 taps, *SD* = 17.3) than girls did [*M* = 9.77, *SD* = 11.2; *t*(28) = 3.02, *p* = 0.005, *d* = 0.96], but girls tapped as frequently as boys during the filler game. This pattern of results is consistent with reliable sex difference in self-regulation reported in the research literature – specifically, that young males have lower self-regulation than females do (Diamond and Taylor, 1996; Kochanska et al., 1996; Silverman, 2003; Matthews et al., 2009).

Two-year-olds learned 2.33 words on average (*SD* = 0.84 word), ranging from 0 to 4 words. This is a relatively low response rate, given prior evidence that children of this age and younger can reliably learn to associate numerous novel labels with objects depicted on computer and video screens (e.g., Golinkoff et al., 1987; Schafer and Plunkett, 1998; Werker et al., 1998; Scofield et al., 2007; Spiegel and Halberda, 2011). A negative (albeit non-significant) tendency was observed for children who tapped more during instruction to make fewer correct responses on the word-learning task. However, there was also substantial variability in the amount of tapping during instructions, and some 2-year-olds who regulated their tapping still had trouble learning the words. Therefore, this preliminary result suggests an important area for future research: identifying other factors that along with self-regulation might contribute to early learning from touchscreens.

The presence of “unsupportive, incidental, inconsiderate hotspots” (Zucker et al., 2009) in the form of the engaging butterflies filler task between word-learning trials may help explain why toddlers were not more successful in identifying the named objects, since the physical response needed to select the target (tapping) was the same response that was encouraged by the filler task. App designers and parents might be alert for and consider how unregulated tapping behavior (possibly engendered by the app itself) could distract from learning goals, especially for young boys.

#### Four- and Five-year-old-children

The older children were significantly more successful at inhibiting tapping during the app instructions and labeling (4-year-olds: *M* = 6.14 taps, *SD* = 11.6; 5-year-olds: *M* = 2.76 taps, *SD* = 4.16) compared to the 2-year-olds [*M* = 19.2 taps, *SD* = 17.0; *F*(2,74) = 13.3, *p* < 0.001]. Thus, our results are in line with reports from cross-sectional and longitudinal research that self-regulation (controlling one’s actions when required by the situation) increases across the preschool period (Gerstadt et al., 1994; Diamond and Taylor, 1996; Kochanska et al., 1996; Carlson, 2005; Garon et al., 2008). Children of all ages were motivated to tap (shown by statistically equivalent tapping during the butterfly filler task (e.g., 2-year-olds: *M* = 7.77 taps, *SD* = 7.70, 5-year-olds: *M* = 4.40 taps, *SD* = 5.89) but older children could better inhibit their tapping during instruction. These results highlight the particular struggle that very young children have in inhibiting their tendency to tap during moments when they are instructed to wait and listen, such as during teaching moments.

As expected, the older age groups responded correctly on significantly more of the four novel word learning trials (4-year olds: *M* = 3.00 words, *SD* = 3.83; 5-year-olds: *M* = 3.36 words, *SD* = 0.76) than the youngest group did [*M* = 2.33 words, *SD* = 0.84; *F*(2,74) = 11.0, *p* < 0.001]. A trial-by-trial analysis revealed that all age groups responded successfully on the first novel word trial, and the 5-year olds responded correctly on all trials. In contrast, the 2- and 4-year-olds’ responses dropped to chance level on the second trial. In some other recent word learning studies, children have shown more robust learning on earlier trials, a kind of “primacy effect” (Horst and Samuelson, 2008; Horst et al., 2010; Zosh et al., 2013). One possibility is that the 2- and 4-year-old children, having succeeded on Trial 1 and then tapped eagerly in the butterfly filler task that followed, did not analyze word learning Trial 2 sufficiently to notice what had now changed (the new object identities and label) that might require a new solution rather than a reflexive response (Aguiar and Baillargeon, 2003). Having experienced two trials, however, some children might extrapolate that certain elements changed across trials, and therefore required focused attention. In fact, the 4-year-olds reliably identified the named target object on the last two trials. The play break with toys that followed Trial 2 may have allowed the 4-year-olds to return and engage with Trials 3 and 4 of the word learning app task less reflexively and more analytically – in Aguiar and Baillargeon’s terms, to realize that “a significant change has been introduced that renders [retrieval of a previous solution] inappropriate” (p. 278). In contrast, the 2-year-olds’ word learning remained at chance. Given the challenge that some 2-year-olds had in self-regulation and inhibiting their tapping behavior, it may not be surprising that this age group had difficulty focusing on how to use their taps to respond thoughtfully on the later trials, when prepotent (i.e., dominant) responses had been set in motion (Garon et al., 2008). Similarly, young preschool-aged children in prior research had particular difficulty on later trials of executive function tasks requiring focused attention and inhibitory control (Diamond and Taylor, 1996; Gerardi-Caulton, 2000).

A challenge for app design pointed out by Study 1 is the use of a prepotent response (tapping) to assess learning in very young children. Additionally, a more stringent test of word learning is needed than choosing and tapping a novel object on the screen once. Language researchers point out that at minimum, some kind of transfer or generalization task is needed to more clearly show word learning (Werker et al., 1998). Several research teams have provided evidence that “fast mapping” between labels and objects may be only the beginning of really understanding how words refer to entities in the world (Axelsson and Horst, 2013; Bion et al., 2013).

In Study 2, we further examined the effect of both child characteristics and app design on children’s learning. Previous research has highlighted the particular benefit of haptic, touch-based exploration (Bara et al., 2007) and particular technology enhancements in e-books (Takacs et al., 2015) for low-SES children’s learning. According to recent surveys, SES status is no longer an impediment to touchscreen experience (Smith, 2013; Kabali et al., 2015). Also, we wanted to follow up on the sex differences that emerged in self-regulation between boys and girls in Study 1. Therefore, in Study 2, we probed whether particular kinds of interaction benefitted lower- and/or higher-SES boys’ and/or girls’ learning.

Regarding app design, we compared the effect of tapping a named object on the screen to a less common, possibly more challenging and engaging behavior: *dragging* an on-screen object. There were at least two possible outcomes of this comparison. On the first account, screen tapping is such a well-practiced, intuitive behavior for most young children that it requires few cognitive resources to carry out. Tapping on relevant/informative areas of the screen promoted learning for at least some preschoolers in recent touchscreen studies (Choi and Kirkorian, 2016; Kirkorian et al., 2016), so tapping may be effective for children of a particular age, sex, and/or SES. An alternative possibility is that the less common, more distinctive, and more motorically challenging behavior of dragging would require children to focus attention to successfully drag the named object, and possibly help them learn its name, similar to the way that distinctive or attention-focusing interactions with a screen promoted adults’ learning in previous research (Sapkota et al., 2013; Schwartz and Plass, 2014). In a new app designed for this study, dragging was a functional behavior that fit the requirements of the cover story (to get objects “across a river”), which might make the event (and object) more memorable. However, if dragging the object turned out to be too challenging or cognitively demanding for our participants, this requirement might impede learning. Based on previous research, we expected interacting with the screen to promote learning better than passively watching events (e.g., Strouse and Ganea, 2016). However, it was also possible that engaging in the prepotent tapping response would be less effective than watching for at least some preschool children. Therefore, in Study 2 we compared the two different active manipulation behaviors to merely watching on-screen events, and looked at the relation between these kinds of interactions and preschool children’s learning and transfer of novel object labels.

## Study 2

### Method

#### Participants

The participants were 182 children from 2 to 4 years of age and their parents. Twelve children were unwilling to complete the task and their data were dropped from analyses, leaving a total of 170 children (*M* = 41.05 months, *SD* = 10.51; 82 males) divided into three age groups (see **Table 1**). Some were recruited through state birth records (*N* = 52) and others through local daycare centers and preschools (*N* = 118). Participants were randomly assigned to one of three conditions, with the caveat that sex and mean age were equated across condition as much as possible: *Watch* condition (*N* = 58, *M* = 40.6 months, *SD* = 10.6 months); *Tap* condition (*N* = 60, *M* = 40.8 months, *SD* = 10.5 months); *Drag* condition (*N* = 52, *M* = 41.8 months, *SD* = 10.5 months). The children were from families with various ethnic backgrounds: parents identified their child as White (62%), Black or African–American (25%), Asian (3%), Biracial or mixed race (5%), or chose not to disclose their child’s race (5%). Socioeconomic status (SES; parents’ highest completed education levels) ranged from high school diploma or less (7.1%) to at least some graduate or professional training (44.1%).

**Table 1:** Mean (standard deviation) age (in months) for the three age groups by gender in Study 2.

	Male	Female
	*n* = 28	*n* = 21
2-year-olds	27.2	28.0
	(3.56)	(3.70)
	*n* = 45	*n* = 39
3-year-olds	42.3 (3.67)	43.1 (2.82)
	*n* = 15	*n* = 22
4-year-olds	55.4	55.3
	(3.35)	(3.59)


#### Materials

**FIGURE 2:**
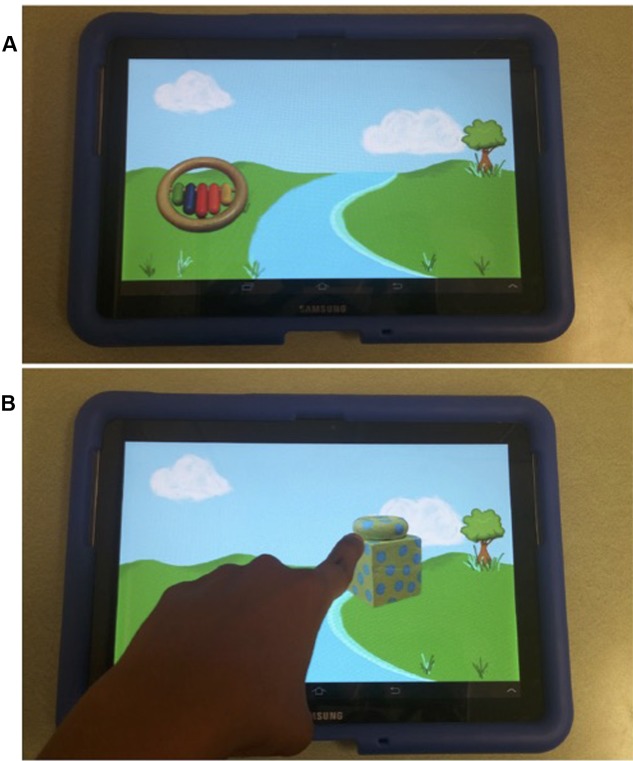
**One pair of novel objects for Study 2:** **(A)** during labeling; and **(B)** after labeling, during a dragging interaction.

A new word-learning app, programmed for us by an undergraduate engineering student, was displayed on a 10.1″ Samsung Galaxy Tab 2 with a 21.7 cm × 13.6 cm active screen area (see **Figure 2**). The app was programmed to automatically record the location, frequency, and time of the participant’s taps or drags on the screen. In the new app, the same familiar and novel objects were labeled as in Study 1. Real, 3D versions of the objects shown on the tablet were also used along with a plastic bucket to contain them. Parents completed a short survey about family demographics and their child’s media use.

#### Procedure

Children played the word-learning app at the lab after participating in a separate, unrelated study not involving a tablet, or in a quiet room of their daycare center or preschool. Depending on condition, they either watched (*watch* condition) or played (*tap* and *drag* conditions) the word learning game on a touchscreen tablet held for them by the experimenter. An assistant unobtrusively coded the interaction while watching over the child’s shoulder. As in Study 1, the first two pairs of objects were familiar animals. These trials served to familiarize the child with the game, but children’s responses were not analyzed. The last four (test) trials involved the novel objects and object labels used in Study 1.

#### Game Play

The app opened with an introductory screen narrated in a voiceover: “Let’s play a game!” The introduction of familiar and novel objects was the same for participants in all conditions. To begin a trial, the app introduced each of a pair of objects, which appeared on a white background one at a time. During the familiarization trials, each animal appeared in turn and was labeled three times. During the test trials, the novel target object was labeled and the distractor object was called “this one.” For example, the target object appeared on the screen, accompanied by the narration, “Look at the Dax! See the Dax? Isn’t the Dax neat?” Then the target object disappeared and the distractor object appeared with the narration, “Look at this one! See this one? Isn’t this one neat?” Again, the object pairings were kept consistent, but the order in which the pairs appeared and which novel object of the pair served as the target were counterbalanced across participants. Whether the target or distractor object appeared first was counterbalanced across the trials.

After a pair of familiar or novel objects was introduced, the app entered the interactive portion. Against a cartoon backdrop of a field bisected by a river (**Figure 2**), one of the pair of objects appeared on the left and the app narrator told the child, “[The ____ /This one] needs to get to the other side of the river.” Then, depending on condition (and using carrier phrases of equal length in each condition), the app instructed the child either to: (1) “*Watch* [the ____ /this one] move across the river!”; (2) “*Tap* [the ____ /this one] and it will move!”; or, (3) “*Drag* [the ____ /this one] and it will move!” Following this instruction and the child’s response, when each object reached the far side of the river it was labeled one final time: “Yay! [The ____ /This one!]” followed by a rewarding chime sound.

The next screen offered a test of the child’s word learning. The pair of objects appeared against a white background, in different locations than during labeling. The narrator asked about the labeled object, “Where is [the ____ ]?” Regardless of the child’s choice (touch response), the app continued with the two objects appearing in new locations on the screen, and the app narrator asking, “Where is [the ____ ] now?” The child did not receive feedback from the app or experimenter on their selections. Each of the six trials (two with familiar objects and four with novel objects) lasted approximately 1 min and there was no off-topic “filler” task.

After each trial with the tablet, the child was tested using the corresponding 3D objects. The experimenter dumped the pair of objects out of the bucket in front of the child, extended her hand on the midline of her body toward the child and asked the child to place the target object in her hand (e.g., “Can you put the Dax in my hand?”). No feedback was given to the child. This sequence took approximately 20 s and was repeated for each of the six pairings (two familiarization/familiar object trials and four novel object trials).

#### Scoring

The assistant recorded children’s object selections (depicted on the tablet and with the real objects) during the session. Children’s touches of an object in the app also were retrieved from the output log on the tablet. A second coder scored children’s real object selections from videotape of the sessions, resulting in 100% agreement across the two coders. To be considered correct on learning an object label on the tablet, children needed to select the named target object in response to both requests to identify it in the app. These strict criteria aimed to avoid giving children credit for word learning that was merely chance selection. Because transfer from the app (the virtual target object) to the real world (the real target object) depended on learning the information in the app, to receive credit for the real object transfer, children needed to select the target object both times in the app, and then select the real, 3D target object.

Socioeconomic status was measured as the average of the two parents’ (or the sole parent’s) education levels (as reported by parents on the survey); education level tends to reflect SES more accurately than income does in our population. In our relatively educated sample, the data were grouped into three categories, each containing about a third of participants: *low SES* parent education corresponded to some high school through some college education; *middle SES* corresponded to a bachelor’s degree through some graduate work; and *high SES* corresponded to a master’s or doctoral degree. Parents reported children’s exposure to touchscreens as the amount of time (in hours) that their child *actively interacted with a touchscreen* in a typical day, excluding such non-interactive uses as watching movies on a touchscreen device.

### Results and Discussion

Children in all age groups learned some words from the tablet, and sometimes transferred what they learned to the real object (see **Table 2**). We first examined parents’ responses to the survey items and the relation between SES and touchscreen exposure. In the analyses below, the degrees of freedom reflect the inclusion of covariates and some missing data in survey items, such as SES reporting.

**Table 2:** Mean number of words (out of four) learned on the tablet and transferred to the real object for the three age groups in Study 2; standard deviations in parentheses.

	Tablet	3D Transfer
2-year-olds	2.22	1.49
	(1.25)	(1.17)
3-year-olds	3.04	2.68
	(1.06)	(1.28)
4-year-olds	3.03	2.95
	(0.96)	(1.03)


#### Descriptive Statistics: SES

Parents reported their highest attained education level as either a high school diploma or less (7.7% of families), some college work/Associate’s degree (20.6%), Bachelor’s degree (28.2%), some graduate work (4.1%), Master’s degree (22.4%), or Doctoral degree (11.2%); 5.8% declined to disclose their education level. Dividing families into three relatively equal SES groups resulted in 58 children in the *low SES* group, 54 children in the *middle SES* group, and 47 children in the *high SES* group.

#### Touchscreen Exposure

Children across our whole socioeconomic range had prior exposure to touchscreens, but there were intriguing SES differences. Even after controlling for age, there was a significant difference in the amount of time children spent with touchscreens depending on their parents’ education level, *F*(2,148) = 8.38, *p* < 0.001, η^2^ = 0.102. Pairwise comparisons (with age-covaried Bonferroni corrections) revealed that children from lower SES families spent significantly more time with touchscreens per day (*M* = 1.50 h, *SE* = 0.16) than children from both middle SES families (*M* = 0.77 h, *SE* = 0.16; 95% CI [0.18, 1.27], *p* = 0.005) and high SES families (*M* = 0.61 h, *SE* = 0.17; 95% CI [0.32, 1.45], *p* = 0.001). Thus, children from lower SES families spent approximately 90 min per day on touchscreens compared to 35–45 min per day for children from middle-and upper-SES families. In contrast, there was no significant difference in the amount of time children of different SES backgrounds watched television—a result that differed from what has previously been reported (Anand and Krosnick, 2005; Fairclough et al., 2009). Because prior exposure to touchscreens differed across SES, we controlled for these factors in subsequent analyses of word learning.

#### Analysis by Age

Children’s word learning was similar to that found in Study 1 (see **Table 2**). In an initial Analysis of Covariance (ANCOVA), a significant age difference emerged in words learned from the touchscreen app (as measured by children’s responses on the tablet). The age difference remained after controlling for SES and prior touchscreen exposure, *F*(2,145) = 11.0, *p* < 0.001, η^2^ = 0.132. Pairwise comparisons (with a Bonferroni adjustment) indicated that 2-year-old children learned significantly fewer words than 3-year-old children (mean difference of -0.91 word, 95% CI [-1.41, -0.42], *p* < 0.001) and 4-year-old children (mean difference of -0.84 word, 95% CI [-1.43, 0.25], *p* = 0.002). Word learning by the 3- and 4-year-old children in our assessment on the tablet was equivalent.

For each age group, we used a paired sample *t*-test to compare children’s word learning on the tablet to their transfer of the label to the actual object. Recall that to receive credit for transferring a word, children needed to have first learned the word on the tablet. Statistically equivalent scores on the tablet and transfer word learning scores would indicate successful transfer of learned words. The results suggest that only the 4-year-old children were proficient in transferring their learning from the tablet to the real 3D objects, with no significant difference between their scores on the two tests of word learning, *t*(36) = 1.78, *p* = 0.083, see **Table 2**. In contrast, there were significant differences in word learning scores on the tablet compared to transferring the labels to the real objects for the 2-year-olds, *t*(48) = 5.98, *p* < 0.001, and 3-year-olds, *t*(83) = 4.34, *p* < 0.001. Because of the clear age difference in word learning, we controlled for age in the remaining analyses.

#### Analysis by Socioeconomic Status (SES)

**FIGURE 3:**
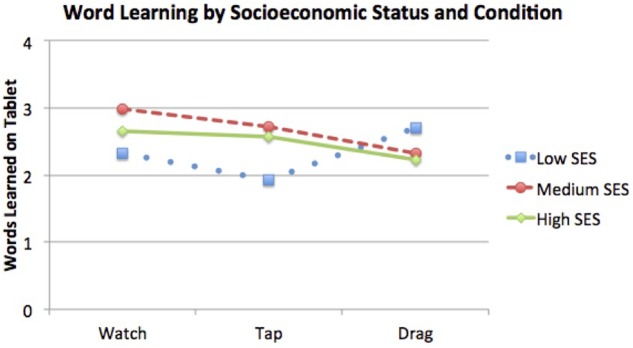
**Number of words learned by children in Study 2 (all ages included) from the three SES groups in the three app interaction conditions, with age covaried.** Children from low SES families (dotted line) learned significantly more words after dragging, compared to tapping, the named object on the screen.

A two-way ANCOVA revealed a significant interaction between condition (watch, tap, drag) and SES (parental education: low, medium, high), on children’s word learning, controlling for age, *F*(4,149) = 2.46, *p* = 0.048, η^2^ = 0.062 (see **Figure 3**). Pairwise comparisons revealed that word learning of low-SES participants, as tested in the app, differed in the interactive tapping and dragging conditions: with a Bonferroni adjustment for multiple comparisons (and controlling for age), participants from low-SES families learned more words by *dragging* the named object (*M* = 3.09, *SE* = 0.26) than by *tapping* it (*M* = 2.22, *SE* = 0.20), with a mean difference of 0.87 word, 95% CI [0.81, 1.66], *p* = 0.025. In the transfer test with the 3D objects, this pattern remained but was non-significant.

#### Analysis by Sex

**FIGURE 4:**
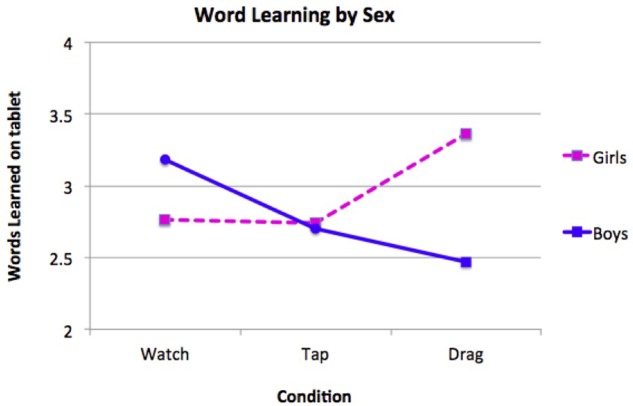
**Word learning (assessed on the tablet) by boys and girls of all ages and SES groups in Study 2.** With SES and age covaried, girls outperformed boys in the interactive *drag* condition, and boys learned significantly more in the non-interactive *watch* condition than in the interactive *drag* condition.

We also looked for any sex differences in children’s word learning (as assessed on the tablet) using a two-way ANCOVA controlling for age and SES. There were no main effects of sex or condition, but a significant sex × condition interaction emerged, *F*(2,151) = 5.09, *p* = 0.007, η^2^ = 0.063 (see **Figure 4**). According to a pairwise comparison (with Bonferroni adjustment for multiple comparisons, controlling for age and SES), girls who dragged named objects learned significantly more of the four words (*M* = 3.36, *SE* = 0.23) than boys did (*M* = 2.47, *SE* = 0.20) – a mean difference of 0.89 word, 95% CI [0.30, 1.49], *p* = 0.004. Another pairwise comparison (with the same adjustments and covariates) indicated that boys in the non-interactive watch condition learned significantly more words (*M* = 3.18, *SE* = 0.20) than boys in the interactive drag condition (*M* = 2.47, *SE* = 0.20), with a mean difference of 0.71 word, 95% CI [0.04, 1.39], *p* = 0.034. Identical (though non-significant) trends for all condition × sex differences emerged when analyzing real object word-learning transfer.

We further investigated girls’ and boys’ word learning on the tablet using follow-up ANCOVAs split by age group, controlling for variance contributed by SES. A significant interaction between sex and condition emerged only for the 3-year-olds, *F*(2,73) = 5.19, *p* = 0.008, η^2^ = 0.125. Pairwise comparisons (with a Bonferroni adjustment and SES covaried) reveal that in the drag condition, 3-year-old girls learned significantly more words (*M* = 3.98, *SE* = 0.38) than 3-year-old boys did (*M* = 2.58, *SE* = 0.25), with a mean difference of 1.40 words, 95% CI [0.49, 2.32], *p* = 0.003. A similar, marginally significant result favoring girls in the drag condition emerged in the 4-year-olds, with a mean difference of 0.97 words, 95% CI [0.002, 1.94], *p* = 0.050). Further, 3-year-old girls learned more words when they dragged the named objects (*M* = 3.98, *SE* = 0.38) than when they simply watched the objects move without interacting with the screen (*M* = 2.78, *SE* = 0.30), although this 1.20-word difference was only marginally significant, 95% CI [-0.003, 2.41], *p* = 0.051. A possible explanation for girls specifically benefiting from the motorically challenging drag condition involves preschool sex differences in fine motor development favoring girls (Moser and Reikerås, 2016).

#### Analysis of Tapping Frequency

A one-way Analysis of Variance (ANOVA) showed a significant condition difference in the amount that children tapped during instruction (when the app narrator was speaking), *F*(2,166) = 14.9, *p* < 0.001. Tukey *post hoc* comparisons revealed that children in the watch condition tapped significantly less than those in the tap condition, with a mean decrease of 28.3 taps, 95% CI [-43.7, -13.0], *p* < 0.001. Children in the watch condition also tapped significantly less than those in the drag condition, with a mean decrease of 33.6 taps, 95% CI [-49.5, -17.7], *p* < 0.001. Specifically, children who *watched* the objects move across the screen tapped on average 10 times during the instruction throughout gameplay, whereas children who *interacted* with the app through tapping or dragging tapped an average of 38 and 44 times, respectively, when they were supposed to be listening. This tapping difference points to one potential mechanism to explain why the children (particularly boys) in the non-interactive watch condition performed better overall than those in the interactive tap condition. That is, tapping during the interaction portions may have primed or elicited additional taps during times that children were instructed *not* to tap, and thus distracted them from encoding the novel object labels. Why, then, did participating in the drag condition (which apparently elicited even more extra taps) still promote learning, at least for girls? Some provisional hypotheses are presented below.

## General Discussion

In the studies reported here, we offer some exploratory insight into how young boys and girls of different ages and family backgrounds interact with touchscreens, and how various types of touch interactions impact learning. Our focus was preschool children’s physical interactions with a touchscreen app, their self-regulation, and their word learning from the screen. We expected that toddlers’ ability to inhibit a dominant response to tap on the screen might be related to their self-regulation as assessed by Carlson’s (2005) snack task, an age-specific standard measure of this aspect of executive function. In Study 1, we purposely designed our simple word-learning app to promote children’s interaction with the screen. To make the app more similar to commercial products that young children use, between the four word-learning trials we inserted a filler task of tapping on butterflies to produce a rewarding chime sound. This off-topic task kept children engaged, but it also may have primed children’s tapping response. We found that 2-year-olds who scored lower on self-regulation tapped more than twice as often while the narrator was labeling objects compared to toddlers with higher self-regulation. Children with better inhibitory control tapped just as frequently as other children during the butterflies screens, but inhibited their tapping during the instruction portions of the app. Compared to the toddlers, older children (4- and 5-year-olds) were significantly better at controlling the tapping response during the app’s instructions and object labeling. This pattern of results fits with cross-sectional and longitudinal evidence that the ability to control one’s actions when required by the situation increases across the preschool period (Diamond and Taylor, 1996; Kochanska et al., 1996; Gerardi-Caulton, 2000; Carlson, 2005; Garon et al., 2008). Our results should alert parents and media professionals to the particular challenge infants and toddlers will face listening to instructions explaining how to play and narration aimed at teaching, when a learning device responds to their touch.

Children of all ages learned the new word on the first trial in Study 1, showing that even 2-year-olds can learn a novel object label from a touchscreen. However, only the older children were reliable word learners over trials. The younger children in both studies had a tendency to tap more over time: they tapped more on the fourth trial than the first trial, although the difference did not reach statistical significance. This tendency echoes the results of studies of inhibitory control in which young children started out following directions, yet ended up responding quickly but inaccurately by the later trials (e.g., Gerstadt et al., 1994; Diamond and Taylor, 1996; Gerardi-Caulton, 2000). In Study 2, 2- and 3-year-olds learned words as assessed within the app, but the oldest children alone (4-year-olds) were proficient at transferring the new object labels to the actual, 3D objects when tested immediately afterward.

Word-learning tasks in which a recorded voiceover labels a close-up of a single object on a laptop (Scofield et al., 2007) or one of a pair of objects on a video or computer screen (e.g., the “preferential looking paradigm”—Golinkoff et al., 1987; Spiegel and Halberda, 2011) are relatively common. However, language researchers point out that making initial word-object associations is not the same as forming an enduring, rich understanding of a word that allows a child to generalize that word’s meaning to novel exemplars (Werker et al., 1998; Horst and Samuelson, 2008; Axelsson and Horst, 2013; Bion et al., 2013; Zosh et al., 2013).

The current results are in line with a general “transfer deficit” that has been reported in many previous studies with screen media including touchscreens (e.g., Barr, 2010, 2013). An important take-away message is that even when young children “get the answer right” within an app, adult support may be needed for children to apply educational information to the world outside the screen (Barr, 2010, 2013; Troseth et al., 2016). When possible, research investigating children’s learning from touchscreen apps should include 3D transfer tasks to measure children’s generalization of learning.

In Study 2, we compared the effect of children’s *tapping* on labeled objects to get them to move “across the river,” *dragging* those objects to move them, or merely *watching* the object move on the screen. There were no overall main effects for which behaviors led to the best word learning. However, there were intriguing interactions involving the learning of children from lower- versus higher-SES families, and of girls compared to boys.

Participants from lower-SES families (where parent reported attaining “some high school” to “some college”) learned more (3 of 4 words) by dragging named objects than by tapping objects to get them to move (just over 2 words). Parent survey data hints at a potential explanation. Children from our lower SES families spent, on average, 90 min per day with touchscreens—at least twice as long as children from the middle- and high-SES groups did. Given our lower-SES children’s relatively abundant touchscreen experience, screen tapping may have been an especially well-practiced, dominant response that was less distinctive than dragging. Typically, lower-SES children’s fine motor development is delayed compared to that of more advantaged children (Piek et al., 2008; Miquoelote et al., 2012; Aiman et al., 2016; Comuk-Balci et al., 2016). However, one contributor to fine motor development (often related to SES) is access to and experience with play materials (Miquoelote et al., 2012). Compared to low-SES groups in prior research, the children in our lower-SES group may have differed in important ways (e.g., many attended a high-quality preschool for low-income families) or their ample exposure to touchscreens may have trained up the specific fine motor abilities needed to interact with the screen.

Dragging was likely to be a relatively novel screen behavior, a more challenging fine motor skill than tapping (requiring focus to accomplish). As a relatively distinctive behavior, dragging a named object may have been more memorable, and a mental representation of the event easier to retrieve, compared to tapping an object to get it to move (similar to why iconic movements incited deeper processing in adults—Schwartz and Plass, 2014). Additionally, the act of dragging objects during the labeling phase was different from the response required during the app-based word-learning *test* (i.e., tapping on the target object when asked to choose). In Aguiar and Baillargeon’s (2003) account of infant problem solving and perseveration, the authors reason that individuals engage in deep processing of a problem when they realize that they cannot apply their previous response to the new problem. Switching between dragging during labeling and tapping during the test may have assisted children who possessed the requisite fine motor skill to respond more intentionally to each new event than in the tapping condition, when the response during labeling and test was the same.

Across SES groups, dragging was more helpful for girls than for boys, especially for the 3- and 4-year-olds. Looking across conditions, 3-year-old girls learned more after dragging named objects (nearly all had perfect scores) than after passively watching the objects move. Boys, on the other hand, learned more in the non-interactive watch condition than in the interactive drag condition. A partial explanation for this sex difference may be more advanced fine motor development in girls during the preschool years (Comuk-Balci et al., 2016; Moser and Reikerås, 2016). Compared to tapping an object, dragging it also is likely to require greater focused attention, monitoring of success, and repair of failures—behaviors that depend on executive function skills such as inhibitory control and selective attention. Earlier development of such self-regulatory behaviors in girls compared to boys (e.g., Kochanska et al., 1996, 2000; Silverman, 2003; Matthews et al., 2009) may explain why the older preschool girls in our research were able to benefit from the dragging response.

Dragging is merely one example of a behavior that, at least for some preschool-aged children, appears to be challenging enough to promote focused attention, while not being too difficult in terms of motor skills. In research with adults, Schwartz and Plass (2014) had participants drag a virtual object (such as an eraser) on a thematically related background (e.g., a blackboard) as an “iconic” movement related to the meaning of a to-be-learned phrase (e.g., *erase the blackboard*). For adults, enacting a *meaningful* action promoted better memory than if they merely clicked and the object moved on its own. Iconic movement served to recruit conceptual information (about erasers and blackboards) and offered multi-sensory retrieval cues for recalling the target phrase after a delay. Dragging was contextually relevant within the app storyline of the object needing to get across the river. This meaningful context for the action of dragging may have served to focus sustained attention on the named target object, helping children with the requisite fine motor control and ability to focus to remember its label at the test.

We had expected tapping on named objects to promote more learning than watching without interacting, but such was not the case for our participants. In previous research with adults and children, tapping or clicking on a relevant item increased learning compared to merely watching an item move (Sapkota et al., 2013) or tapping elsewhere on the screen to advance the action (Choi and Kirkorian, 2016; Kirkorian et al., 2016). Similarly, children who pressed a button on a computer to get videos to play learned more than children who watched non-interactive video (Lauricella et al., 2010).

An analysis of children’s tapping behavior in Study 2 by condition is illuminating. Across the duration of the app, children who *watched* the objects “move across the river” tapped a total of only 10 times, on average, during the narrator’s instruction (when objects were being labeled), whereas children who *tapped* on objects to make them move went on to tap on average four times as often during instruction, when they were supposed to be listening. Interacting with the screen by tapping to move the labeled object may have primed children’s prepotent tendency to tap reflexively on the screen, which then carried over into periods of instruction, possibly distracting children from focusing on the words. In the context of tapping across all app segments, tapping the target objects may have been more *reflexive* (automatic) than *reflective* (with deep processing of the object’s identity). Thus, tapping as an interactive behavior may not have effectively directed children’s attention.

Children in the drag condition also tapped four times as often during instruction compared to children in the watch condition. The fact that children who watched without interacting tapped comparatively seldom may help explain why boys in particular (with their less-advanced self-regulation ability compared to girls) learned better when they watched than when they interacted with the screen by dragging: they were more likely to learn from touchscreens in situations that did not prime them to touch the screen (and thus reduced the chances of distraction).

An exception to this pattern was found in low-SES children in the watch condition. There was no overall interaction between SES and condition in the amount that children tapped on the screen. However, with age covaried, a marginal difference in tapping frequency emerged between the low SES group and the other groups, which is specifically apparent in the *watch* condition, in which the lower SES children tapped much more (18.7 taps) compared to the middle SES (6.39 taps) and the high SES groups (7.11 taps). Based on the connection between tapping and self-regulation in Study 1, we might infer that the low-SES children in Study 2 were exhibiting lower self-regulation, a finding commonly reported in the literature (see Sarsour et al., 2011, for a review). However, when app gameplay involved directed interaction (the request to *tap* or *drag* objects), there was no difference in the number of taps elicited between the SES groups.

Dragging the named object did help the girls—despite the extra tapping that was engendered by interacting with the screen. As mentioned earlier, for children with better self-regulation (and fine motor control), dragging recently labeled objects may have been optimally challenging so as to focus attention on the object being moved. Thus, dragging named objects may have resulted in memorable event representations that promoted word learning, despite the fact that interacting with the screen also promoted children’s tapping when the narrator was offering the object labels. Similarly, dragging seemed to help the lower-SES children, experienced touchscreen users, to focus on and learn the words, whereas watching events on the touchscreen engendered excessive tapping.

The research reported here has several limitations. Although we measured 2-year-olds’ self-regulation in Study 1, we did not collect this data from the other children. Future independent assessment of older girls’ and boys’ self-regulation and fine motor skills will help to support or refute our suggestions of mechanisms underlying the differential benefits of the various kinds of interactions. Our word-learning apps were not commercial products, so were limited in many ways compared to touchscreen app products developed to teach language. Additionally, as is suggested by parents’ responses to our media survey (which differed from published survey results from a few years earlier—e.g., Anand and Krosnick, 2005; Fairclough et al., 2009), “children’s experience with media” is a moving target; exposure to new products and technology will continue to change the skills, expectations, and responses that children bring to the experience of learning from educational technology.

The current research involved a learning app that directed children what to do. Thus, it does not answer questions about how children learn during self-directed exploration on a touchscreen. In intentional exploratory learning, a person decides to examine a new object, sight, or sound, instead of being told or guided to do so. Intrinsically motivated actions (*volitional movements)* allow a child to choose how they wish to engage with material, and intrinsic motivation is important in creating engaged learners (Hirsh-Pasek et al., 2015). Previous research has shown that when adults explored a virtual on-screen environment, periods of active, intrinsically motivated exploration (compared to times when the person was not moving) were marked by increased responses in areas of the hippocampus involved in learning and memory. Furthermore, those increased responses predicted learning, memory, and later performance accuracy (Kaplan et al., 2012). Self-directed actions on a touchscreen might similarly promote children’s active processing and learning.

The message emerging from research, including the current studies, is that interactivity from touchscreens is a double-edged sword: on the one hand, haptic engagement (including touches on a responsive screen) can direct attention and focus and contribute to learning in adults (Smith and Olkun, 2005; Sapkota et al., 2013) and children (Huber et al., 2015; Choi and Kirkorian, 2016; Kirkorian et al., 2016). On the other hand, research indicates that interactivity in the form of hotspots and games can actually distract from learning (Takacs et al., 2015) due to the need for a child to “task switch” or disengage from the interactive feature and selectively re-focus on educational content (Fisch, 2000; Sweller, 2005; Mayer and Moreno, 2003; Bus et al., 2015). Given young children’s limited ability to regulate their own attention and actions, developers of children’s media must think strategically about using interactivity in ways that benefit, rather than hinder, learning. In a recent study with toddlers, for instance, Strouse and Ganea (2016) zeroed in on differences between more and less helpful interactivity for this age group: very simple interaction, such as having to tap on an object to turn the page, helped toddlers learn a word, but if the tap elicited an engaging but “off-topic” visual and auditory reward, children’s learning suffered.

A hopeful finding in the current research was that lower-SES children (defined as children whose parents had less education) learned better in the challenging condition that required dragging named objects, compared to the condition that called for a tap response. Well-designed interactive technology holds great promise for giving children from less advantaged families additional engagement with educational content, particularly as touchscreen devices have now become prevalent across all income levels (Kabali et al., 2015). Differences in how girls and boys (or children of different ages) learn best can be met by making digital technology customizable (e.g., including a parent control panel) so parents and educators can tailor an app by choosing an interactive style that best fits the child. Developers can utilize play testing to observe how children with different characteristics engage with the app, and try to accommodate as many types of beneficial interaction styles as possible.

Our studies highlight a significant but perhaps overlooked aspect of children using educational technology: that *how* a child interacts with an app may be as important as the app’s content in determining how much a child learns. Parents and industry experts should consider a child’s age, sex, level of self-regulation, screen experience, and the physical requirements of engagement with touchscreens when designing, choosing, and studying educational apps for children.

## Ethics Statement

This study was carried out in accordance with the recommendations of and approval of the Vanderbilt University Institutional Review Board. All parents of children who participated gave written informed consent, and all participating children gave verbal assent, in accordance with the Declaration of Helsinki. Children were given the option to cease participation at any time if they did not want to continue.

## Author Contributions

CR-J and GT oversaw both studies. CR-J, GT, and CD contributed original ideas for both studies. CD led data collection for Study 1, AM led data collection for Study 2. CR-J, GT, AM, and CD wrote the manuscript.

## Conflict of Interest Statement

The authors declare that the research was conducted in the absence of any commercial or financial relationships that could be construed as a potential conflict of interest.
